# Spatially resolved visualization of reprogrammed metabolism in hepatocellular carcinoma by mass spectrometry imaging

**DOI:** 10.1186/s12935-023-03027-0

**Published:** 2023-08-24

**Authors:** Bangzhen Ma, Yang Zhang, Jiwei Ma, Xinguo Chen, Chenglong Sun, Chengkun Qin

**Affiliations:** 1 Shandong Provincial Hospital, Shandong University, Jinan, 250021 China; 2 Shandong Provincial Hospital Affiliated to Shandong First Medical University, Jinan, 250021 China; 3 School of Pharmaceutical Sciences, Qilu University of Technology (Shandong Academy of Sciences), Jinan, 250014 China; 4 Key Laboratory for Applied Technology of Sophisticated Analytical Instruments of Shandong Province, Shandong Analysis and Test Center, Qilu University of Technology (Shandong Academy of Sciences), Jinan, 250014 China

**Keywords:** Hepatocellular carcinoma, MALDI-MS, Spatially resolved imaging, Metabolites, lipids

## Abstract

**Background:**

Metabolic reprogramming refers to tumor-associated metabolic alterations during tumorigenesis and has been regarded as one of the most important features of cancer. Profiling the altered metabolites and lipids in hepatocellular carcinoma with spatial signature will not only enhance our understanding of tumor metabolic reprogramming, but also offer potential metabolic liabilities that might be exploited for hepatocellular carcinoma therapy.

**Methods:**

We perform matrix-assisted laser desorption/ionization-mass spectrometry imaging (MALDI-MSI) analysis on both hepatocellular carcinoma xenograft mouse model and hepatocellular carcinoma patients. Discriminatory metabolites that altered during the development of hepatocellular carcinoma are screened and imaged in xenograft mouse model and are further validated in 21 hepatocellular carcinoma patients.

**Results:**

We discover stepwise metabolic alterations and progressively increasing metabolic heterogeneity during the growth of hepatocellular carcinoma. Arginine and its metabolites spermine and spermidine, choline and phosphatidylcholine metabolism, and fatty acids were found to be significantly reprogrammed in hepatocellular carcinoma tissues.

**Conclusions:**

The spatially resolved profiling of the metabolites and lipids in highly heterogeneous hepatocellular carcinoma tissue will contribute to obtaining precise metabolic information for the understanding of tumor metabolic reprogramming.

**Supplementary Information:**

The online version contains supplementary material available at 10.1186/s12935-023-03027-0.

## Background

Hepatocellular carcinoma is one of the most common malignant tumors worldwide, ranking sixth in incidence among malignant tumors and third in cause of death from tumors [1]. Reprogrammed metabolism is regarded as an important feature of tumors, which helps to elucidate the mechanism of its occurrence and provides potential targets for clinical treatment [2–4]. Disturbed energy metabolism in cancer cells can alter many biologically relevant metabolic pathways, such as cell proliferation and regulation, and as a common feature of all cancer cells, altered metabolism has always been an important direction of cancer research [5, 6].

Performing metabolomic studies on tumor tissues can not only characterize the metabolic signature of tumors, but also help to identify potential metabolic markers associated with tumor initiation, progression, and metastasis [7]. Recently, researchers have successfully carried out liquid chromatography-mass spectrometry (LC-MS) based metabolomic analysis on hepatocellular carcinoma tissues and have made significant progress in profiling the metabolic signatures of hepatocellular carcinoma [8, 9]. For example, Xu’s group performed LC-MS based nontargeted tissue metabolomics analysis on fifty pairs of hepatocellular carcinoma samples and matched normal tissues, and found that the glycolysis, gluconeogenesis, and β-oxidation were upregulated and tricarboxylic acid cycle and Δ-12 desaturase were downregulated in hepatocellular carcinoma [10]. Ferrarini et al. explored the metabolomic characteristics of tumor and nontumor tissues from 40 hepatocellular carcinoma patients using LC-MS and gas chromatography (GC)-MS platforms. A total of 18 metabolites in tricarboxylic acid (TCA) cycle, glycolysis, purines, and lipid metabolism pathways were screened as key molecules related to the development of hepatocellular carcinoma [8]. Liu et al. discovered that the levels of DL-3-phenyllactic acid, L-tryptophan, glycocholic acid and 1-methylnicotinamide in hepatocellular carcinoma tissues were significantly higher than those in healthy controls by performing LC-MS based untargeted metabolomic profiling [11]. However, it should be noted that hepatocellular carcinoma tumor tissue is highly heterogeneous, which means that the distributions of metabolites in hepatocellular carcinoma tissue is also heterogeneous. Traditional metabolomics studies carried out based on LC-MS technique requires experimental processes such as tissue homogenization, metabolite extraction, and chromatographic separation, in which, unfortunately, information on the spatial distributions of metabolites in heterogeneous tumor tissues is completely destroyed.

Mass spectrometry imaging (MSI) allows in situ analysis of metabolites in tissue sections without disrupting their spatial distribution characteristics [12–18]. Matrix-assisted laser desorption/ionization-mass spectrometry imaging (MALDI-MSI) and desorption electrospray ionization-mass spectrometry imaging (DESI-MSI) are currently the two most commonly used mass spectrometry imaging techniques for the visualization of metabolites in biological tissues [19–24]. By performing MSI analysis on highly heterogeneous tumor tissues, the researchers successfully screened tumor in situ diagnostic markers and identified potential therapeutic targets through metabolic pathway analysis, paving way for deeper understanding of tumor molecular characteristics and better tumor therapy [25–31].

**Fig. 1:**
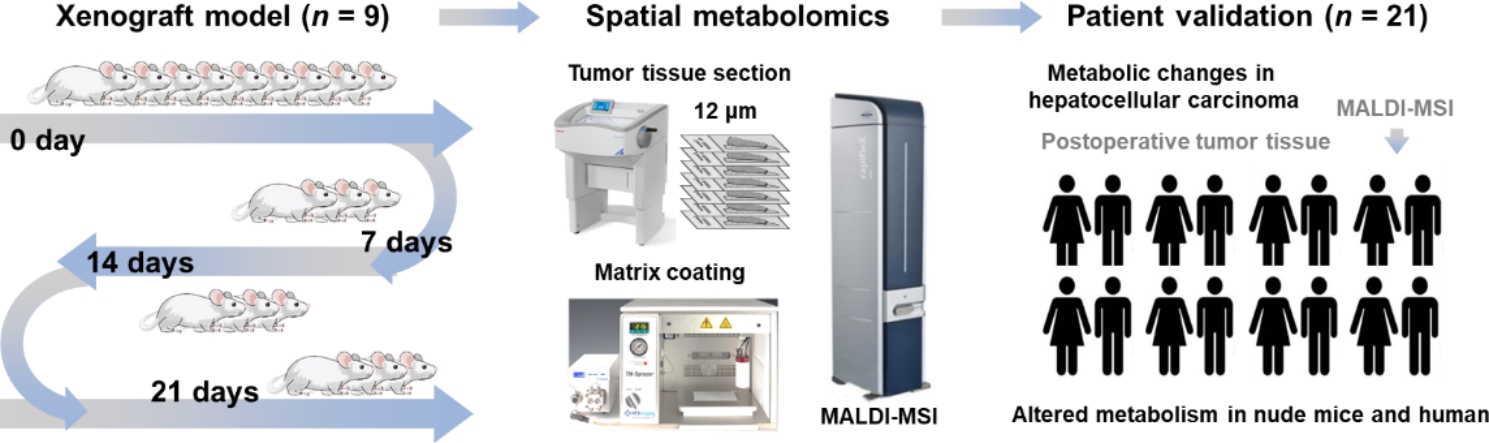
Schematic illustrations for spatially resolved visualization of reprogrammed metabolism in hepatocellular carcinoma by mass spectrometry imaging

In this study, we conduct MALDI-MSI analysis on the tumor tissues of hepatocellular carcinoma xenograft mouse model and hepatocellular carcinoma patients. Discriminatory metabolites that altered during the growth and progression of hepatocellular carcinoma were first screened and imaged in xenograft mouse model and then were further validated in postoperative human hepatocellular carcinoma tissues. The design of this work is illustrated in Fig. 1.

## Methods

### Chemicals and reagents

1,5-Diaminonaphthalene (1,5-DAN) was purchased from Shanghai Aladdin Biochemical Technology Co., Ltd (Shanghai, China). HPLC-grade acetonitrile (ACN) and methanol (MeOH) were afforded by Merck (Darmstadt, Germany). O.C.T. embedding agent was provided by Sakura Finetek Japan Co., Ltd. (Tokyo, Japan). The indium tin oxide (ITO)-coated slides were purchased from Bruker (Daltonics, Billerica, MA). The SUPERFROST PLUS slides were provided by Thermo Fisher Scientific (Waltham, MA, USA). Experimental water was provided by Wahaha Co., Ltd. (Hangzhou, China).

### Cell culture

A human hepatocellular carcinoma cell line (HepG2 cell) was purchased from the Shanghai Cell Bank, Chinese Academy of Sciences. The cell culture medium was MEM with 10% fetal bovine serum (FBS), streptomycin (100 µg/mL), and penicillin (100 µg/mL). Cells were cultured at 37 °C, 5% CO_2_ atmosphere.

### Establishment of in vivo experimental animal models

The human hepatocellular carcinoma cell line HepG2 was stored in liquid nitrogen until use. The healthy male BALB/c nude mice (4 weeks old) were purchased from Spelford Biotechnology Co., Ltd (Beijing, China) and housed in the animal house of the Provincial Hospital of Shandong University. All animal husbandry and experiments were performed in accordance with the policy guidelines established by the Institutional Animal Husbandry and Use Committee of Shandong University Provincial Hospital. The human hepatocellular carcinoma cell line HepG2 was cultured in cell culture flasks, and after routine recovery, HepG2 cells were centrifuged, washed twice with PBS, and resuspended in PBS. 100 µL of cell suspension, with a total cell count of approximately 1 × 10^6^, was injected into the subcutaneous space of the foreleg to establish an ectopic transplantation tumor model.

Nude mice were randomly divided into three groups of three mice each when the subcutaneous tumor volume grew to 100 mm^3^. The nude mice were housed in the SPF environment of the Animal Experiment Center of the Provincial Hospital of Shandong University, and the subcutaneous tumor sampling of one group of nude mice was per-formed every 7 days. The anesthetic drug aphrodisiac, 280 mg/kg, was administered intraperitoneally once before sampling, and all mice were anesthetized and their tumor specimens were taken. All mice were anesthetized and their tumor specimens were taken. After sampling, the mice were killed by inhalation of carbon dioxide. The nude mice were disposed of according to laboratory regulations. The subcutaneous tumors were stored in a -80 refrigerator for freezing and then analyzed by MALDI mass spectrometry. The animal experiment was conducted at Laboratory Animal Research Center of Shandong Provincial Hospital with approval by the Animal Care and Use Committee of Shandong Provincial Hospital (No. 2022-040).

### Collection of human hepatocellular carcinoma tissue sample

This study was performed according to the Declaration of Helsinki and Good Clinical Practice and approved by the Ethnical Committee of Shandong Provincial Hospital (No. 2022-069). This study included hepatocellular carcinoma tissue from 21 patients who underwent surgery. The post-operative hepatocellular carcinoma tissues were immediately placed in dry ice and then transferred to a -80 °C refrigerator.

### Preparation of tissue section

Human and mouse hepatocellular carcinoma tissues were embedded in Tissue-Tek O.C.T. agent, and then were cut into continuous twelve-micron tissue sections using a cryostat microtome (CryoStar NX50 NOVPD, Thermo, Bremen, Germany). For each hepatocellular carcinoma tissue, one of the hepatocellular carcinoma frozen tissue sections was subjected to H&E staining, and two of the hepatocellular carcinoma frozen tissue sections were subjected to MALDI mass spectrometry imaging analysis in positive and negative ion modes, respectively.

### Matrix coating

1,5-DAN was selected as the MALDI matrix for hepatocellular carcinoma analysis. 1,5-DAN (2.5 mg/mL) in ACN/H_2_O (70:30, *v*/*v*) was sprayed onto the tissue sections by a HTX TM-Sprayer™ (HTX Technologies, Carrboro, USA). The flow rate of MALDI matrix solution was set to 75 µL/min. Nozzle nitrogen pressure was set to 10 psi, and nozzle temperature was set to 55 °C. A total of 12 sprays of matrix solution were performed on the surface of hepatocellular carcinoma tissue sections. The nozzle travel speed was set to 80 cm/min. The space between the two spray tracks was set to 0.3 cm. The nozzle-to-slice distance was set to 4 cm.

### MALDI-MS imaging and data analysis

RapifleX MALDI Tissuetyper™ TOF/TOF MS (Bruker Daltonics, Billerica, MA) was used for MALDI-MS imaging of hepatocellular carcinoma tissue sections. MALDI-MSI analysis experiments were performed in positive and negative ion mode in the range of *m/z* 70-1000. The spatial resolution for the MALDI-MS imaging experiments was set to 100 μm. LasAtten was optimized according the ion intensities and resolutions. The MS images of metabolites were constructed using SCiLS Lab 2018b software (GmbH, Bremen, Germany). The ion intensities and in situ data analysis were also calculated using SCiLS Lab 2018b software.

## Results

### Visual characterization of the global metabolic profile of hepatocellular carcinoma during growth

**Fig. 2:**
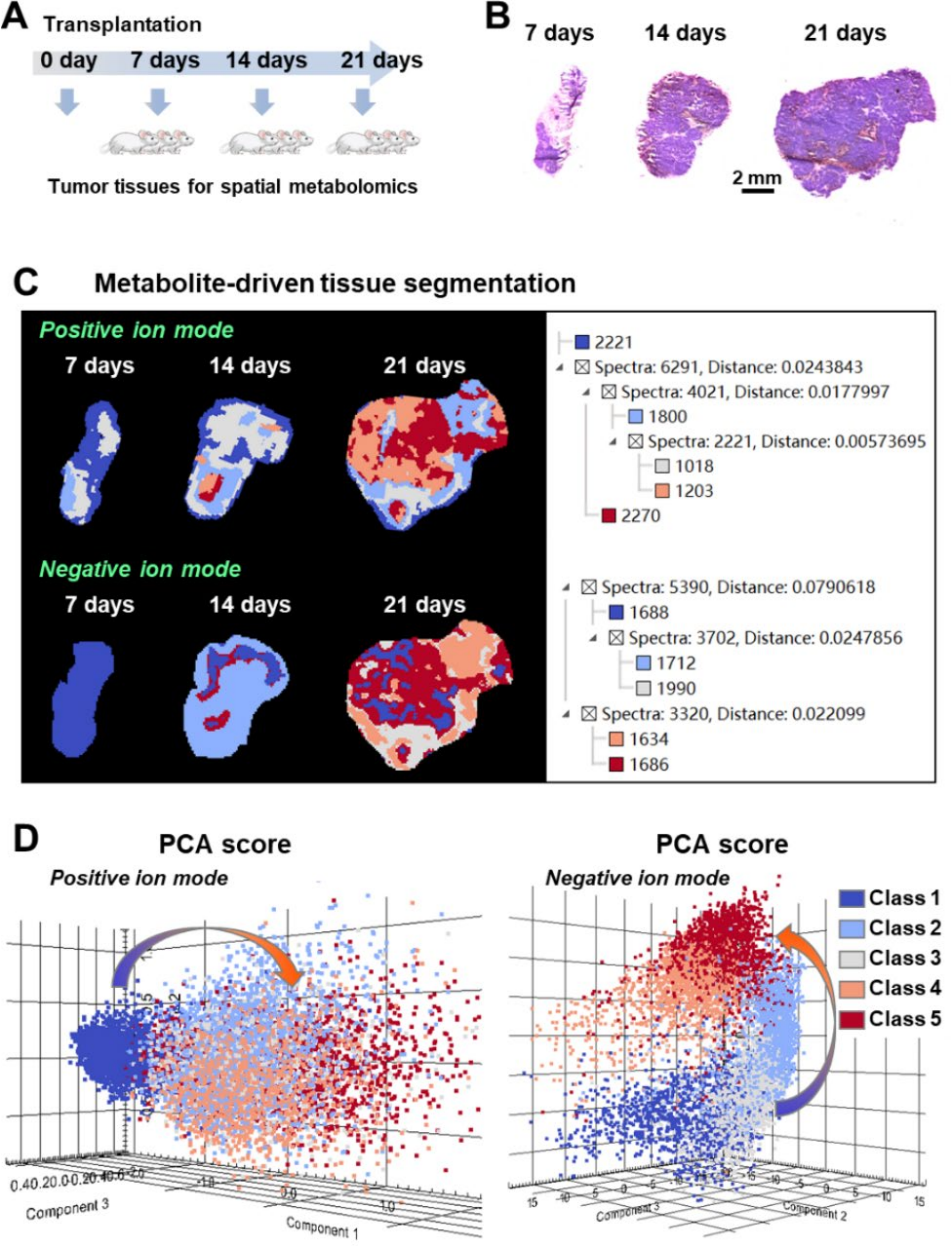
Imaging of the global metabolic profile of transplanted hepatocellular carcinoma tumors after 7, 14 and 21 days of growth. (**A**) Collection of tumor tissues at different growth stages. (**B**) H&E stain imaged of hepatocellular carcinoma tissue sections. (**C**) Data-driven tissue segmentation analysis. (**D**) Principal component analysis (PCA) for transplanted tumors

We first establish xenograft model in nude mice with human hepatocellular carcinoma HepG2 cells. A total of nine nude mouse were used to place xenografts in this study. Tumor tissues from three nude mouse were taken on the 7th, 14th and 21st days post transplantation (Fig. 2A). The tumor tissue sections at different growth processes were prepared for H&E staining. Figure 2B shows the typical H&E stain images of hepatocellular carcinoma tissue sections at different growth stages. Then, we performed MALDI-MS imaging analysis on two adjacent tissue sections of H&E-stained sections in positive and negative ion detection modes, respectively.

After MALDI-MS imaging experiment, we carried out data-driven segmentation analysis on hepatocellular carcinoma tissue sections according to the region-specific MALDI-MS fingerprints. In the data-driven segmentation analysis, different tumor regions with similar MALDI-MS fingerprints are grouped and assigned specific colors, meaning that tissue regions assigned the same color have very close metabolic characteristics. Figure 2 C shows the data-driven segmentation result of transplanted tumors after 7, 14 and 21 days of growth. The results suggest that not only the tumor volume increases gradually during the growth of hepatocellular carcinoma, but also its metabolism is found to be significantly altered. In both positive and negative ion modes, 7-day transplanted tumor tissue sections were given more of cool blue color, while 21-day transplanted tumor were given more of a hot red color. In addition, we found that 21-day transplanted tumor tissue sections exhibited a variety of different colors, suggesting that tumors exhibit significant metabolic heterogeneity at this stage of growth. Moreover, we extracted MALDI-MS data from distinct segmentation regions and per-formed unsupervised principal component analysis. The results showed that the metabolic characteristics of distinct segmentation regions were different and showed stepwise alterations with the growth of tumors (Fig. 2D).

### Screening and imaging of altered metabolites during the growth of hepatocellular carcinoma

#### Arginine metabolism pathway is altered in hepatocellular carcinoma growth

**Fig. 3:**
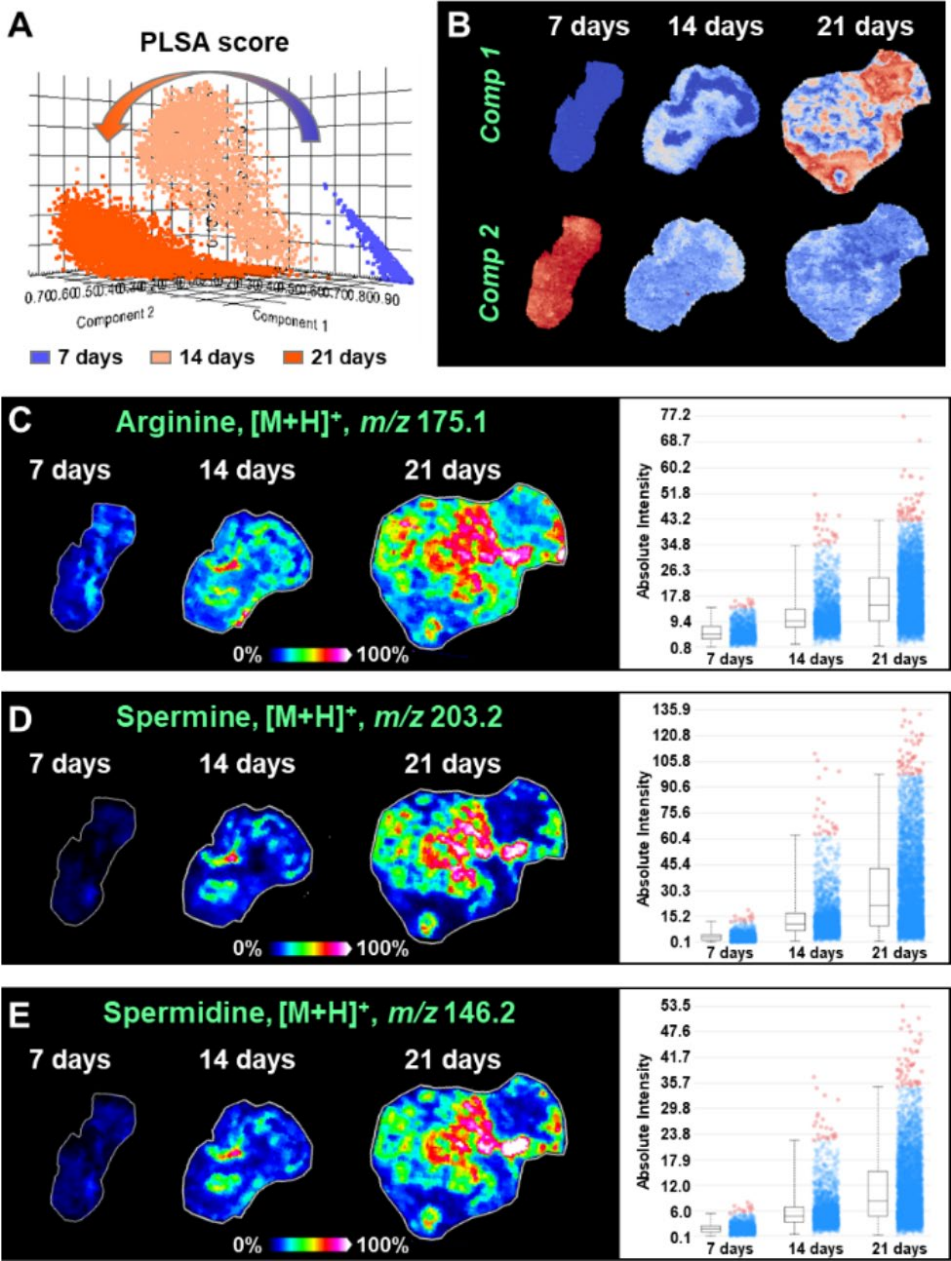
(**A**) Data-driven PLSA analysis for transplanted hepatocellular carcinoma tumors after 7, 14 and 21 days of growth. (**B**) Two main PLSA components that distinguish different hepatocellular carcinoma tissues. (**C-E**) MS images and levels of arginine, spermine, and spermidine in different transplanted tumors. Comp, component

Probabilistic latent semantic analysis (PLSA) model was further used for multivariate statistical analysis and screening of differentially expressed metabolites in distinct hepatocellular carcinoma tissues. PLSA score plots show a clear separation among 7-day, 14-day, and 21-day transplanted tumor tissue, indicating significant metabolite alteration during the growth of hepatocellular carcinoma (Fig. 3A). On this basis, we compressed all metabolites into two fundamental components to characterize the main trends of altered metabolites. Component 1 represents metabolites that are significantly upregulated during the growth of hepatocellular carcinoma, while Component 2 indicates a class of metabolites that are downregulated during the growth of hepatocellular carcinoma in nude mice (Fig. 3B).

By screening discriminatory metabolites among different transplanted tumors, we found that the expression of arginine ([M + H]^+^, *m/z* 175.1) increased gradually during the growth of hepatocellular carcinoma (Fig. 3C). Arginine can be further metabolized to spermine and spermidine under the catalysis of ornithine decarboxylase (ODC), spermidine synthase (SRM), and spermine synthase (SMS). Here, the MALDI-MSI data suggest that the levels of spermine ([M + H]^+^, *m/z* 203.2) and spermidine ([M + H]^+^, *m/z* 146.1) also exhibited upregulated expressions during the growth of hepatocellular carcinoma (Fig. 3D and E).

### Cholines and phosphatidylcholines are altered during the growth of hepatocellular carcinoma

**Fig. 4:**
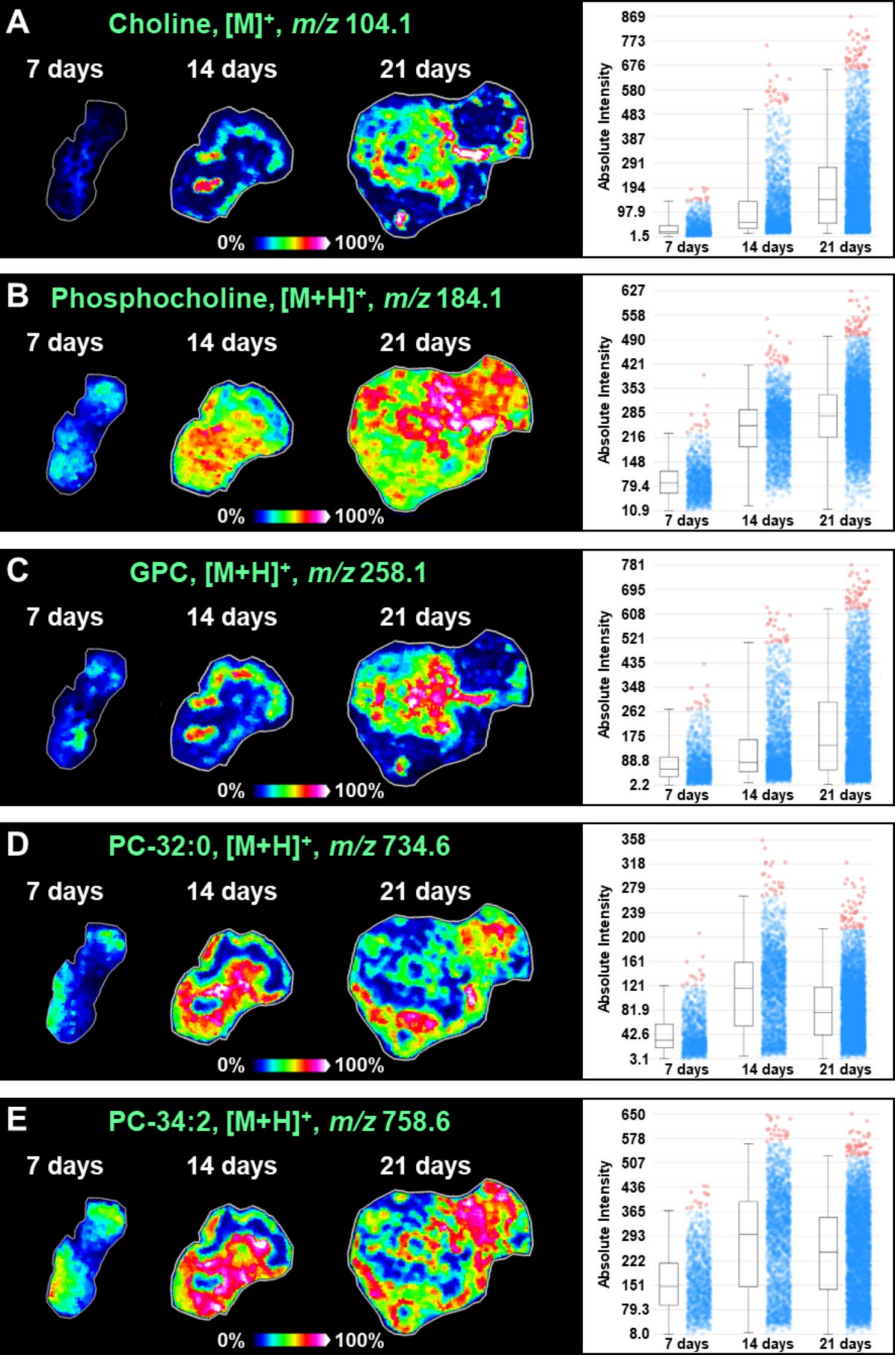
(**A**) MS images and levels of choline in different transplanted tumors. (**B**) MS images and levels of phosphocholine in different transplanted tumors. (**C**) MS images and levels of glycerophosphocholine (GPC) in different transplanted tumors. (**D**) MS images and levels of PC-32:0 in different transplanted tumors. (E) MS images and levels of PC-34:2 in different transplanted tumors

Choline is an important component of phosphatidylcholines (PC), and it also regulates cellular lipid metabolism and transmethylation metabolism. In this study, we discovered that choline ([M]^+^, *m/z* 104.1) exhibited an upregulated expression in the growth of hepatocellular carcinoma (Fig. 4A). Choline can combine with phosphoric acid to form phosphocholine, which can further generate PC under the catalysis of choline kinase (CHKA) and diacylglycerol cholinephosphotransferase (CPT). Coincidentally, the level of phosphocholine ([M + H]^+^, *m/z* 184.1) was found to be also upregulated with the growth of hepatocellular carcinoma (Fig. 4B). In addition, many PCs such as PC-32:0 ([M + H]^+^, *m/z* 734.6) and PC-34:2 ([M + H]^+^, *m/z* 758.6) also show a gradually increasing expression trend (Fig. 4D and E). However, it should be noted that compared to choline and phosphocholine, PCs increase less with tumor growth. Glycerolphosphocholin (GPC) is an important product of phosphatidylcholine metabolism, and our MALDI-MSI results indicate that GPC ([M + H]^+^, *m/z* 258.1) expression also increases gradually during the growth of hepatocellular carcinoma (Fig. 4C).

### The expressions of fatty acids are altered during the growth of hepatocellular carcinoma

**Fig. 5:**
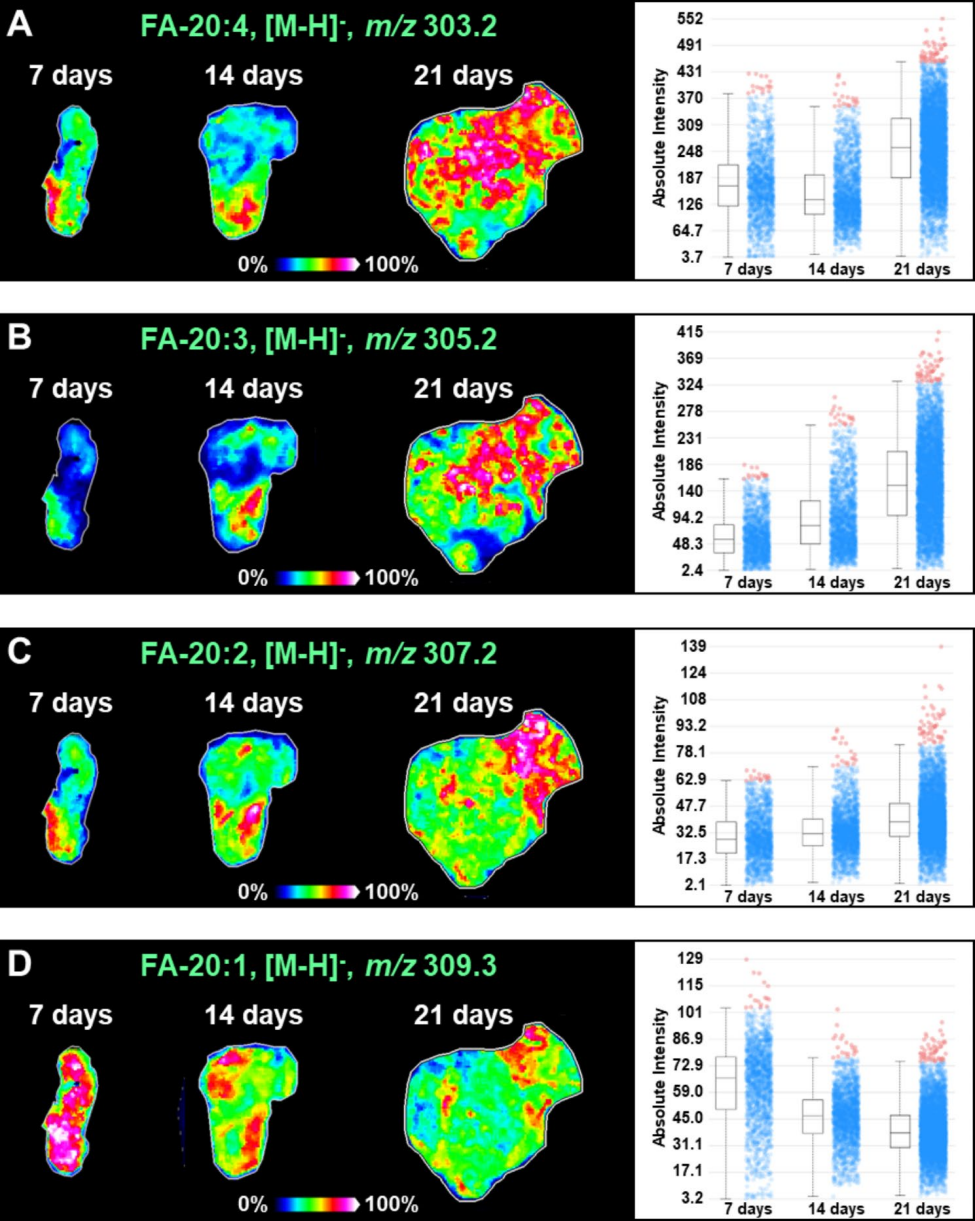
MS images and levels of fatty acids including FA-20:4 (**A**), FA-20:3 (**B**), FA-20:2 (**C**), and FA-20:1 (**D**) in different transplanted tumors

Here, we also discovered that fatty acid (FA) undergoes significant metabolic reprogramming during hepatocellular carcinoma progression. However, it should be noticed that polyunsaturated FAs showed different expression trends from monounsaturated and saturated FAs. Polyunsaturated FAs, such as FA-20:4 ([M-H]^−^, *m/z* 303.2) and FA-20:3 ([M-H]^−^, *m/z* 305.2), exhibited gradually up-regulated expression in 7-day, 14-day, and 21-day transplanted tumor tissues (Fig. 5A and B). The level of FA-20:2 ([M-H]^−^, *m/z* 307.2) did not differ significantly in 7-day, 14-day, and 21-day transplanted tumor tissues (Fig. 5C). As for FA-20:1 ([M-H]^−^, *m/z* 309.2), its expression showed a gradual decrease with tumor progression (Fig. 5D).

### Other altered metabolites in hepatocellular carcinoma

In addition, we screened out some other metabolites that are altered during the growth of transplanted tumors. Malic acid is an important intermediate metabolite in the tricarboxylic acid (TCA) cycle, and it was found that the expression of malic acid exhibited a continuous increase with tumor growth (Fig. S1). Glutamate can enter the TCA cycle to provide energy for cell growth. Here, we discovered that glutamate also showed an upregulated expression in the growth of hepatocellular carcinoma (Fig. S2). Phosphorylation is essential for aerobic oxidation and anaerobic glycolysis of glucose in cells, and the MALDI-MSI result suggest that the level of glucose-phosphate is significantly increased in 21-day transplanted tumor than that in 7-day and 14-day transplanted tumors (Fig. S3).

### Verification of discriminatory metabolites screened from xenograft mouse model on human postoperative hepatocellular carcinoma tissue

**Fig. 6:**
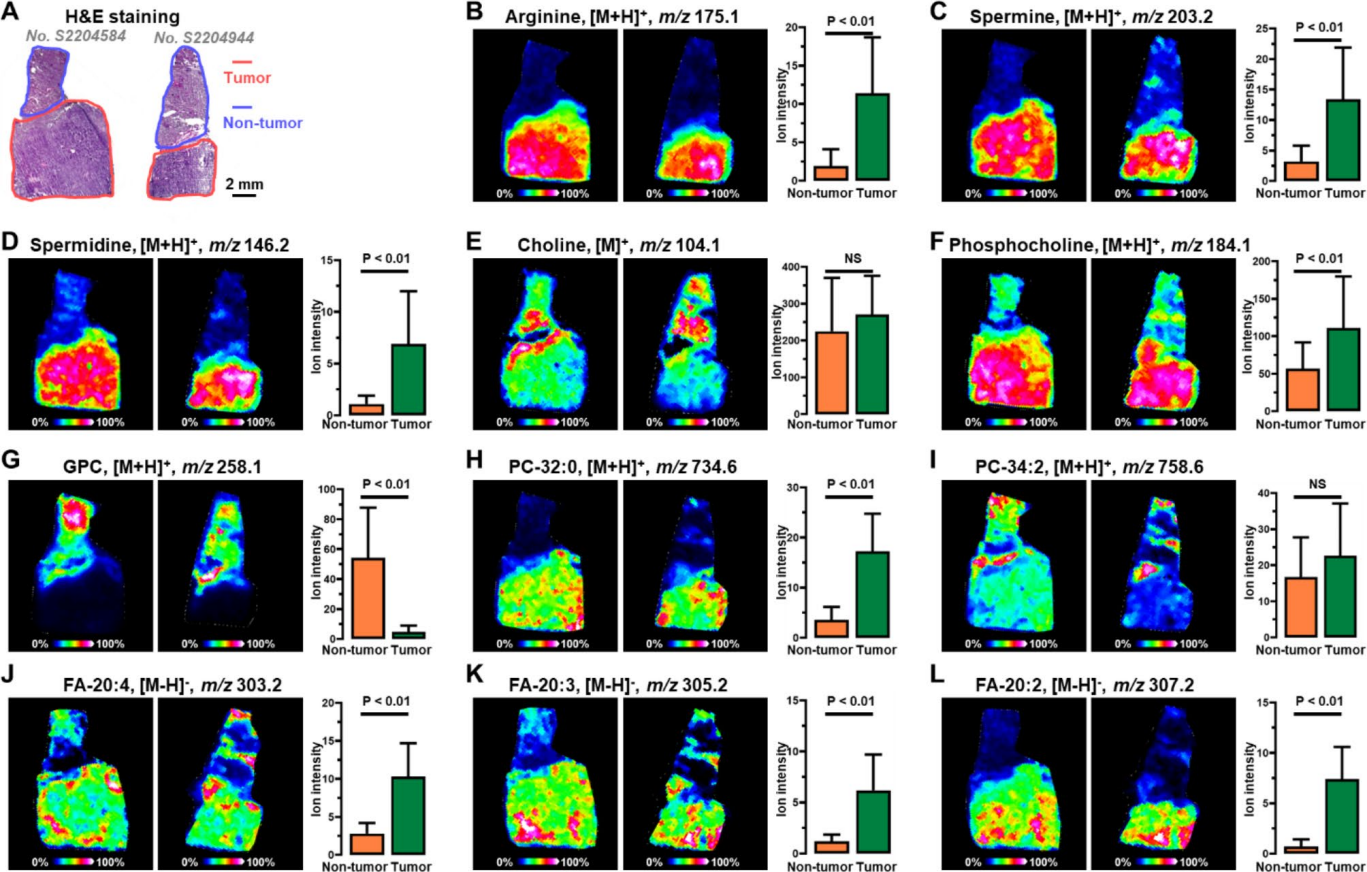
(**A**) H&E stain images of human postoperative hepatocellular carcinoma tissue sections. (**B-L**) MS images of arginine, spermine, spermidine, choline, phosphocholine, glycerophosphocholine, PC-32:0, PC-34:2, FA-20:4, FA-20:3, and FA-20:2 in human postoperative hepatocellular carcinoma tissues. NS, No significant differences

By performing MALDI-MSI based spatial metabolomics analysis on hepatocellular carcinoma xenograft mouse model, we successfully screened for metabolites associated with hepatocellular carcinoma progression. Further, we performed MALDI-MS imaging analysis on 21 postoperative human hepatocellular carcinoma tissues. The postoperative hepatocellular carcinoma tissue samples contained both tumor tissue and adjacent non-tumor tissue. Figure 6B and L demonstrate the MALDI-MS images of arginine, spermine, spermidine, choline, phosphocholine, glycerophosphocholine, PC-32:0, PC-34:2, FA-20:4, FA-20:3, and FA-20:2 in representative human hepatocellular carcinoma tissue sections. Arginine, as well as spermine and spermidine, increased gradually during the growth of hepatocellular carcinoma in xenograft mouse model. In human hepatocellular carcinoma tissue, the expression of arginine, spermine, and spermidine were found to be significantly higher in tumor regions than in non-tumor regions (Fig. 6B and D). The expressions of choline and phosphatidylcholines are altered during the growth of hepatocellular carcinoma in xenograft mouse model. The MALDI-MSI results also suggest that the metabolism of choline and phosphatidylcholines were reprogrammed in tumor tissue compared to surrounding non-tumor tissue. However, choline and GPC increased with tumor growth in xenograft mouse model, whereas in human hepatocellular carcinoma tissue, the expression of choline was not significantly upregulated in tumor tissue, and the expression of GPC was downregulated in tumor tissues (Fig. 6E and G). Similar to the xenograft mouse model, the levels of phosphocholine and PC-32:0 were significantly higher in tumor tissue than in paired non-tumor tissue (Fig. 6F and H). There was no significant difference in the expression of PC-34:2 in tumor and surrounding non-tumor tissue (Fig. 6I). In xenograft mouse model, we found that some FAs showed abnormal expression during tumor progression. Polyunsaturated FAs represented by FA-20:4 and FA-20:3 showed progressively higher expression during the growth of transplanted tumors, while FA-20:1 showed little change and FA-20:1 expression gradually decreased. In human hepatocellular carcinoma tissue, we discovered that polyunsaturated FAs, monounsaturated FAs, and saturated FAs all exhibited significantly higher expressions in tumor tissue than surrounding non-tumor tissue (Fig. 6J and L, and Fig. S4).

## Discussion

Tumor cells need to absorb energy and nutrient compounds from the surrounding environment to maintain their growth and progression. In addition, there is growing evidence showed that tumor cells can also reprogram their metabolic networks to increase energy supply and biomolecule synthesis, and the altered metabolism is recognized as an important feature of tumors. Exploring how reprogrammed tumor metabolic networks affect tumor growth is an important prerequisite for discovering potential metabolic targets to better cancer treatment [32, 33]. However, it should be noted that tumor tissues are highly heterogeneous and the spatial distribution characteristics of metabolites in different micro-regions of tumor tissues may be completely different. Mass spectrometry imaging can direct analyze metabolites in tissue sections, visualize the spatial distributions and relative contents of different metabolites in distinct micro-regions of tumors, thus provide technical support to accurately and spatiotemporally characterize the metabolic features of tumors during their development. In this study, we carried out MALDI-MS imaging analysis on both hepatocellular carcinoma xenograft mouse model and hepatocellular carcinoma patients. Discriminatory metabolites that altered during the growth and progression of hepatocellular carcinoma were first screened and imaged in xenograft mouse model and then were further validated in postoperative human hepatocellular carcinoma tissues. Overall, the metabolic profile of the transplanted tumor shows stepwise alterations during its growth. In addition, we also found that the metabolic heterogeneity of the tumor increases as it grows. Metabolite-driven segmentation analysis indicate that the transplanted tumor exhibited slight heterogeneity after 7 days of growth, but the metabolic heterogeneity significantly increased after 21 days of growth (Fig. 2C).

However, there are some limitations in the visualization of metabolites in biological tissues using MALDI-MSI. For example, when performing MALDI-MSI analysis, we must use matrix, and the presence of matrix-related ions is an important factor restricting the imaging of low molecular weight metabolites. The use of multiple MSI techniques is a good means to improve the coverage of metabolite visualization [34]. In addition, the resolution of the MALDI-MSI used in this study is 100 μm. Whereas most of the cells in the heterogeneous tumor microenvironment have a diameter of about 10 μm, which makes it difficult to visualize and analyze metabolites at the single-cell level.

Polyamines are a class of metabolites containing two or more amino groups, mainly including spermine, spermidine and putrescine. Polyamines play important roles in DNA synthesis and replication, regulation of gene transcription and translation, cell proliferation and apoptosis, and maintenance of cell membrane stability [35]. Previous studies have shown that disorders in the metabolism of polyamines and polyamine synthases are closely related to the development of tumors, and targeting the altered polyamine metabolism is a rational approach for tumor treatment. In this study, we found that polyamines including spermine and spermidine were significantly upregulated in human hepatocellular carcinoma tissues, and exhibited a gradually increase during the growth of hepatocellular carcinoma in xenograft mouse model (Figs. 3 and 6). This is consistent with previous reports of significantly elevated levels of polyamines in other cancers such as esophageal and gastric cancers [22, 36].

Choline and phosphatidylcholine showed significant alterations in both hepatocellular carcinoma xenograft mouse model and human hepatocellular carcinoma tissue. Choline is an important precursor molecule for the synthesis of phosphatidylcholine, which in turn is an important class of phospholipids in cells. Abnormal phospholipid metabolism is closely related to the occurrence, progression, and metastasis of many tumors [37, 38]. In fact, we found that phospholipids with different carbon chain composition can exhibit different changing trends in hepatocellular carcinoma. Some phospholipids such as PC-32:0 showed significant upregulation in hepatocellular carcinoma tissues, while others such as PC-34:2 showed little difference in tumor and adjacent non-tumor tissues (Figs. 4 and 6). We speculate that this may be due to the different biological roles played by different phospholipids in tumor progression, however, this speculation needs further validation.

Fatty acids are important components of cell membranes and play an essential role in cellular signaling and energy metabolism. In the present study, we found that most of the fatty acids were significantly upregulated in tumor tissue, except for individual fatty acids such as FA-20:2 and FA-20:1, which showed little change or downregulation during the growth of transplanted tumors. This echoes the biological role of fatty acids and the previous studies [39, 40]. During the rapid growth and proliferation of tumor cells, fatty acids are needed for β-oxidation to provide energy and also indispensable for the synthesis of lipids.

In summary, we built hepatocellular carcinoma xenograft model and carried out MALDI-MS imaging analysis on tumor tissues at different growth stages. Combined with multivariate statistical analysis, we successfully screened discriminatory metabolites that significantly altered during the growth of transplanted tumor. Furthermore, we performed MALDI-MSI analysis on 21 human postoperative hepatocellular carcinoma tissues to validate the discriminatory metabolites that screened on xenograft model. We discovered that arginine and its metabolites, spermine and spermidine, were significantly upregulated in tumor tissue of hepatocellular carcinoma, and their ex-pressions showed a continuous increase with tumor growth. The metabolism of choline and phosphatidylcholine are altered during the growth of hepatocellular carcinoma. We also found that the levels of most fatty acids in tumor tissues are significantly higher than those in non-tumor tissues. These findings not only enhance our understanding of hepatocellular carcinoma metabolism, but also provide potential metabolic targets for better cancer treatment.

### Electronic supplementary material

Below is the link to the electronic supplementary material.

Supplementary Material 1: Fig. S1–Fig. S4.

## Data Availability

All data generated and analyzed in this study are available by reasonable request of the corresponding author.
