# Supplementary material for: Spatially resolved visualization of reprogrammed metabolism in hepatocellular carcinoma by mass spectrometry imaging
Source: Cancer Cell Int. 2023 Aug 24;23:177. doi: 10.1186/s12935-023-03027-0 (PMC10464423; doi:10.1186/s12935-023-03027-0)
Supplement: Supplementary file 1 — Supplementary Material 1: Fig. S1–Fig. S4. [file 12935_2023_3027_MOESM1_ESM.docx]

**Supplementary Information**

**Spatially Resolved Visualization of Reprogrammed Metabolism in Hepatocellular Carcinoma by Mass Spectrometry Imaging**

Bangzhen Ma^1^, Yang Zhang^2^, Jiwei Ma^2^, Xinguo Chen^1,2^, Chenglong Sun^3,4,*^ and Chengkun Qin^1,*^

^1^ Shandong Provincial Hospital, Shandong University, Jinan, Shandong,250021, P.R. China.

^2^ Shandong Provincial Hospital Affiliated to Shandong First Medical University, Jinan, Shandong,250021, P.R. China.

^3^ Key Laboratory for Applied Technology of Sophisticated Analytical Instruments of Shandong Province, Shandong Analysis and Test Center, Qilu University of Technology (Shandong Academy of Sciences), Jinan, 250014, China.

^4^ Key Laboratory for Natural Active Pharmaceutical Constituents Research in Universities of Shandong Province, School of Pharmaceutical Sciences, Qilu University of Technology (Shandong Academy of Sciences), Jinan, 250014, China.

**Table S1**. Demographic and characteristics of hepatocellular carcinoma patients.

| Characteristics | Number of patients (*n*=21) | Percentage of patients (%) |
| --- | --- | --- |
| Age(years) |  |  |
| ≤50 | 7 | 33.3 |
| ＞50 | 14 | 66.7 |
| Gender |  |  |
| Male | 19 | 90.5 |
| Female | 2 | 9.5 |
| Tumor stage |  |  |
| Ⅰ | 0 | 0 |
| Ⅱ | 0 | 0 |
| Ⅲ | 13 | 61.9 |
| Ⅳ | 8 | 38.1 |


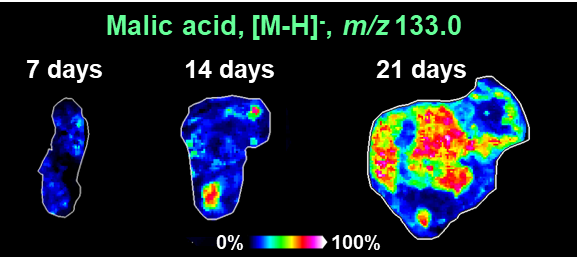


**Fig. S1**. MS images of malic acid in different transplanted tumors.


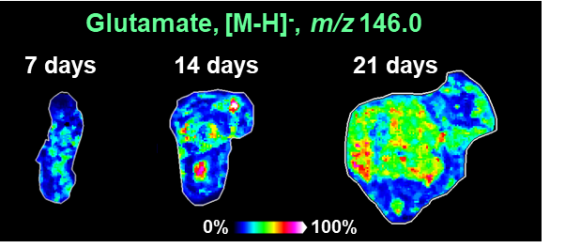


**Fig. S2**. MS images of glutamate in different transplanted tumors.


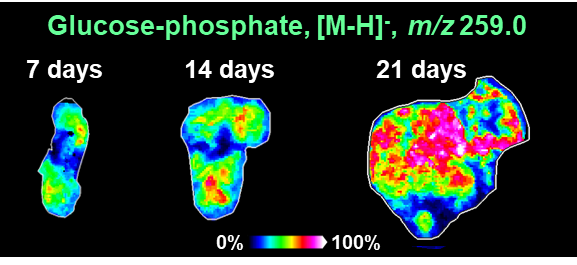


**Fig. S3**. MS images of glucose-phosphate in different transplanted tumors.


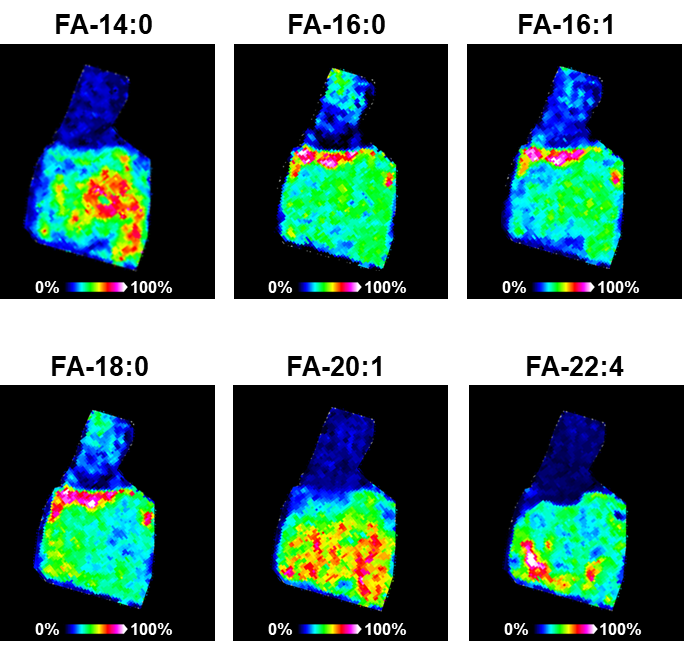


**Fig. S4**. MS images of representative fatty acids in in human postoperative hepatocellular carcinoma tissues.
